# Eye, Ear, Nose, and Throat

**Published:** 1894-11

**Authors:** 


### *SELECTIONS AND ABSTRACTS.*

---

### EYE, EAR, NOSE, AND THROAT.

EDITED BY DUNBAR ROY, A.B., M.D.

### A CASE OF ORBITAL CELLULITIS FOLLOWING TENOTOMY FOR SQUINT.

The above unusual case is reported by Dr. Charles H. May in the July issue of the *Annals of Ophthalmology and Otology*. The patient, a boy six years of age, was operated upon for internal strabismus. The first division of the tendon not correcting the squint, a second operation upon the muscle of the other eye was made under ether anesthesia two months later. For some unknown reason the conjunctival wound from the latter operation became infected and a violent orbital cellulitis was the result. Under careful treatment the eye recovered, and with an excellent result as far as the muscular correction. The doctor was thoroughly antiseptic throughout, and the only way he could account for the infection was its occurrence by means of the wet compresses applied by the family on the evening of the operation when the boy complained of pain.

This case teaches us a very important lesson, and that is, not to make too light of the simple operation for squint. Whenever there is a cut into the conjunctiva a bleeding surface is exposed and an excellent point is afforded for the entrance of the various micro-organisms. For this and other reasons, we have never favored the method which some surgeons have of operating in their office upon a case of strabismus and allowing the patient to return home or to go out about their business without any protection over the eye which has been operated upon. Such a procedure may seem brilliant and be a great advertisement to the ophthalmologist, to be known as the surgeon who has done away with the "old and barbarous methods" of bandaging the eye, and who allows patients to go immediately about their work without any protection over this visual member. While nine such cases may prove successful, the tenth may cause him many a troublesome moment in his endeavors to save this organ from total destruction. It is indeed painful to see the manner in which the poor, ignorant mortals are deluded by these blatant quacks who herald in the secular press the fact that they are the only (self-constituted) surgeons who straighten eyes by a "painless method, without the use of chloroform or ether and allow their patients to go immediately about their business." Shame to the memory of Koller that the fact should not be universally known, that since his introduction of cocaine in '85, painless ophthalmic practice is now a thing of every-day occurrence! Such statements as above quoted might prove "deluding snares" in the wilds of Africa, but in this enlightened age of the nineteenth century, people should cease having their bodies destroyed by these medical vultures.

---

### DENTITION, WITH DISEASES OF THE TEETH AS A CAUSE OF EAR DISEASES.

In March 31st issue, of this year, there appears in the *Medical and Surgical Reporter* the above article by Dr. Laurence Turnbull, of Philadelphia, which is of so much practical value that we reproduce it as fully as we can in a limited space.

"Every physician of experience has found that eruption of the teeth is a cause of earache, followed by pain and a discharge of pus with a laceration of the drum membranes. Especially so when (as frequently happens) the teeth are so impacted that they cannot erupt, if not relieved by lancing the gums. Again, the so-called wisdom teeth, from their reflex irritation and pressure, are a cause of deafness and should be removed. Caries of the teeth, caused by acids and fungi (leptothrix furcatis), is usually attended by both toothache and earache from extension into the ear through the Eustachian tube. If children and adults are of feeble frame and the vitality is lowered by constitutional diseases like syphilis, scrofula, or consumption, the teeth are generally of a chalky white color, brittle or soft, and decay with great rapidity by the action of the leptothrix living on carious stumps, which decay impairs digestion and nutrition; when a cold is taken these stumps being dead, there is almost always an attendant coryza. This affects the ears, causing a running ear, better known by the term otorrhea, but more correctly as an acute otitis media catarrhalis or purulenta. Trophic changes also occur in the Eustachian tube or middle ear with alveolar abscess, or with disease of the antrum of Highmore. The nasal mucous membrane is also a fruitful cause of ear trouble. The tartar, it is stated, protects the tooth substance, but by pressing away the gum and causing absorption of the process, it causes irritation and a loosening of the teeth, and gives rise to changes in color and healthy character of the gums, and the whole buccal mucous membrane; this red condition extending into the throat and ear is often an indication of disease of the bronchial tubes and lungs. In hospital and surgical practice a solution of the permanganate (24 grs. to the ounce) is usually kept on hand as an antiseptic, deodorant, and detergent. Of this solution half a drachm or thirty drops may be added to a pint of boiling water; this can be used in the throat, mouth, nose, and ear when there is a discharge of pus from the meatus. In passing we would simply state our personal experience in regard to dental plates. These should be of gold, platinum, and dark vulcanite. Certain forms of vulcanite, colored by mercury (vermilion), we have found to produce disease of the gums and buccal cavity extending to the ear. In regard to fillings we prefer gold, but there are cases in which gold cannot be used; then a soft material should be used free from acids and mercury.

"The following is a list of some of the diseases of the ear, due in part, or wholly, to reflex irritation of the teeth: Acute and catarrhal inflammation of the middle ear; subacute and chronic purulent inflammation of the middle ear; otitis externa, alveolar abscess and ozena. Sometimes we have found the metal filling forced down into the jaw by too great a pressure of the mallets, which are now used, and in one case such was the cause of profound deafness; hearing was improved after the removal of the pressure. Such a case as this, however, is fairly to be charged to malpractice. No dentist should dream of packing gold or other metal in the apices of the roots; the apices may be closed with other kinds of stoppings (none of which ought to be, nor usually are, so forced as to extrude), and then the body of the root is filled with gold or other material."

### OPTIC NEURITIS AND ATROPHY FROM IODOFORM POISONING THROUGH A BURN.

Such a case is reported by Valude in Soc. d' opht. de Paris, April, 1893.

A boy, aged twelve years, with a deep and extensive burn, treated with iodoform dressings, soon developed hectic fever and suppuration. Valude saw him eight months after the accident, at which time he had vomiting, diarrhea, and headache. The fever which had disappeared shortly before, came on again, and the patient became very anemic. The vision rapidly diminished, so that in a few days the patient became completely blind. When salol was substituted for the iodoform that had been used continuously, all symptoms improved except the amblyopia. Sixteen months after the accident, eleven months after the first symptoms of intoxication, the condition was as follows: Both pupils react promptly; R. V. equals fingers at 10 cm.; L. V. equals fingers at 20 cm.; color perception lost; optic disks white and atrophic, without signs of previous neuritis. The retinal vessels were scarcely narrowed. The onset of amblyopia simultaneously without undoubted symptoms of intoxication, renders it probable that the amblyopia was of the same nature. It is nevertheless not impossible that the amaurosis was due to symptoms on the part of the nervous system that are frequently seen after extensive burns even without intoxication.

